# Supplementary material for: Observation of topological states residing at step edges of WTe2
Source: Nat Commun. 2017 Sep 22;8:659. doi: 10.1038/s41467-017-00745-8 (PMC5610310; doi:10.1038/s41467-017-00745-8)
Supplement: Supplementary file 1 — Supplementary Information [file 41467_2017_745_MOESM1_ESM.pdf]

Description of Supplementary Files

File Name: Supplementary Information
Description: Supplementary Notes, Supplementary Figures and Supplementary Tables

File Name: Peer Review File

Supplementary Note 1. Electronic structure of monolayer 1*T*'-WTe_2

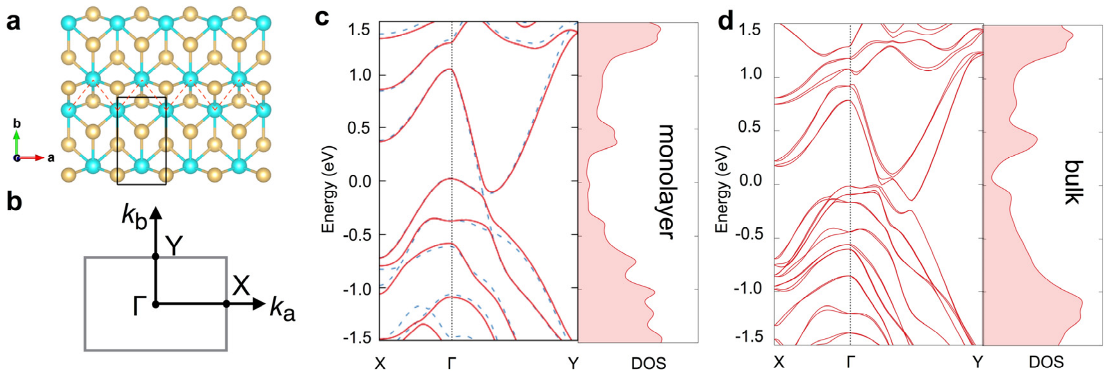

Supplementary Figure 1: Electronic structure of 1*T*'-WTe_2 monolayer. a, The crystal structure of 1*T*'-WTe_2. Its unit cell is indicated with a black rectangle. The zigzag chain of the displaced W atoms is depicted with a red dashed line. **b,** The corresponding Brillouin zone of 1*T*'-WTe_2. **c**, The electronic structure of 1*T*'-WTe_2 (blue dashed line) displays a band crossing slightly below the Fermi level between Γ and Y, which is gapped out by the SOC (solid red line). The corresponding density of states is shown on the right. **d,** Same as **c**, but for the bulk 1*T*'-WTe_2.

The monolayer structure of 1*T*'-WTe_2 shown in Supplementary Figure 1a was taken from its bulk crystal structure and was fully relaxed before the electronic structure was calculated. The bulk electronic structure of 1*T*'-WTe_2 monolayer, shown as the blue dashed line in Supplementary Figure 1c, features the system a gapless semimetal when the SOC is absent. The top of valence bands and the bottom of conduction bands appear separately at Γ and a k-point between Γ-Y. The inclusion of the SOC gaps out the band crossing residing

between Γ and Y, giving rise to the metallic ground state of 1*T*'-WTe_2 monolayer. While, a momentum-dependent Fermi energy can still be found to fully separate the valance from the conduction, which validates the definition of a Z_2 topological invariant in this system that will be discussed in Supplementary Note 4.

Supplementary Note 2. Tunneling spectra of edge state along *a* direction at different locations

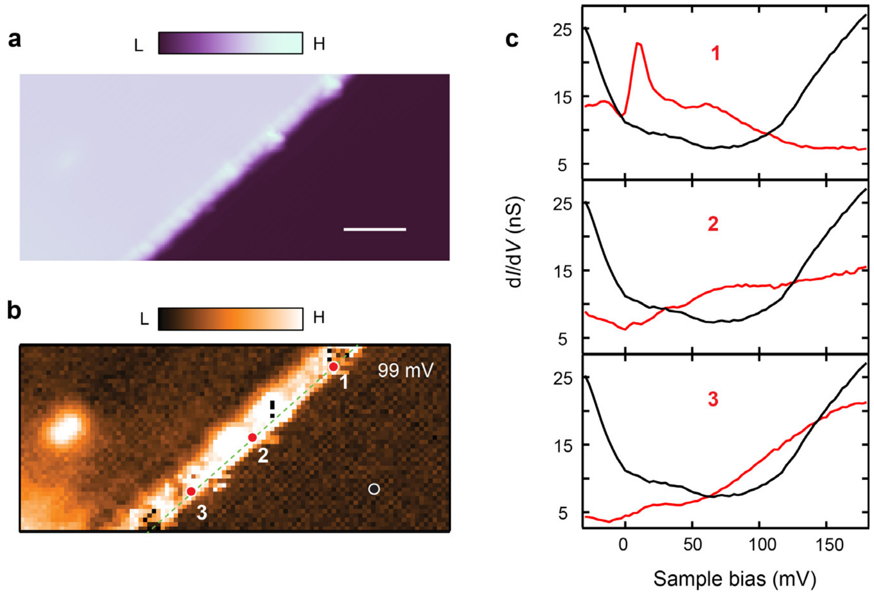

Supplementary Figure 2: Spectra of edge state at different locations of *a*-edge. a, Topography of the single layer high step edge along *a* direction. The scale bar is 5 nm. **b,** Spectroscopic mapping of the step edge in **a** at 99 mV. The data in **a** and **b** are the same as in Fig 2 of main text. **c,** Tunneling spectra (red curves) at different locations of the step edge (red dots in **b**). The spectroscopy (black curve) of the inner terrace (black dot in **b**) is shown for comparison.

Supplementary Note 3. Defect states and their comparison with the edge state

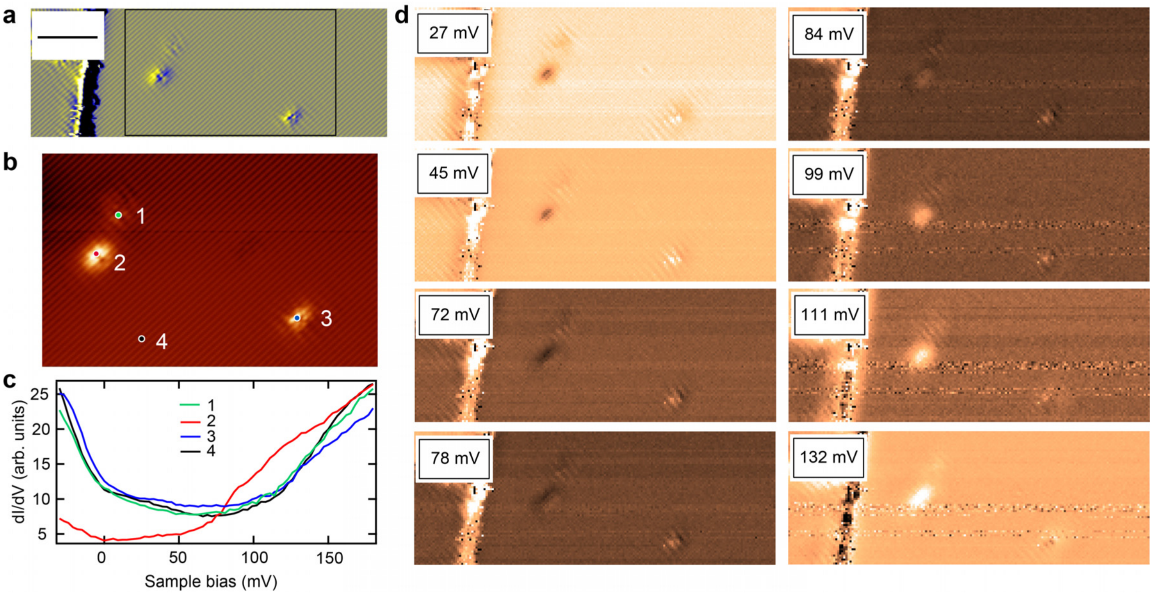

Supplementary Figure 3: Spectra of defects and the comparison of their spectroscopic mapping with the edge state. a, Derivative topographic image of a single layer high step edge and 3 types of frequently observed defects. The step edge is about 38^0 relative to *a* direction. The scale bar is 10 nm. **b**, Enlarged view of the rectangle area in **a** shows the morphology of the defects. Measurement conditions: V_s = 180 mV, I_t = 200 pA. **c,** Tunneling spectroscopy of the defects. While defect 2 exhibits considerable difference compared to the substrate (black curve), defect 1 (green curve) and 3 (blue curve) show no obvious spectroscopic features. It is noted that defect 2 is of the same type as that of Fig. 1 d and e in main text. **d,** Spectroscopic mapping of the step edge and the defects at different voltages. The conductance intensity of the defects show different dependence on voltage compared to the ES, ruling out the possibility of the ES from the defects. Measurement conditions of **c** and **d**: V_s = 180 mV, I_t = 600 pA, V_{mod} = 3.5 mV_{rms}.

To tackle the possibility that the spectroscopic features at the edges are defect induced, we have scrutinized the spectroscopic mapping data shown in Supplementary Figure 3, which show a step edge and three types of typical defects in the system (Supplementary Figure 3b). It is seen from Supplementary Figure 3c that only defect 2 show clear defect state. However, its defect state is different from the edge state. This is evidenced by their difference in spectroscopic intensity with bias (Supplementary Figure 3d). For instance, defect 2 exhibits enhanced (depressed) conductance intensity than the substrate at 132mV (72 and 78mV), when the edge state shows depressed (enhanced) intensity.

Supplementary Note 4. Z_2 topological invariant from topological obstruction and hybrid Wannier charge center

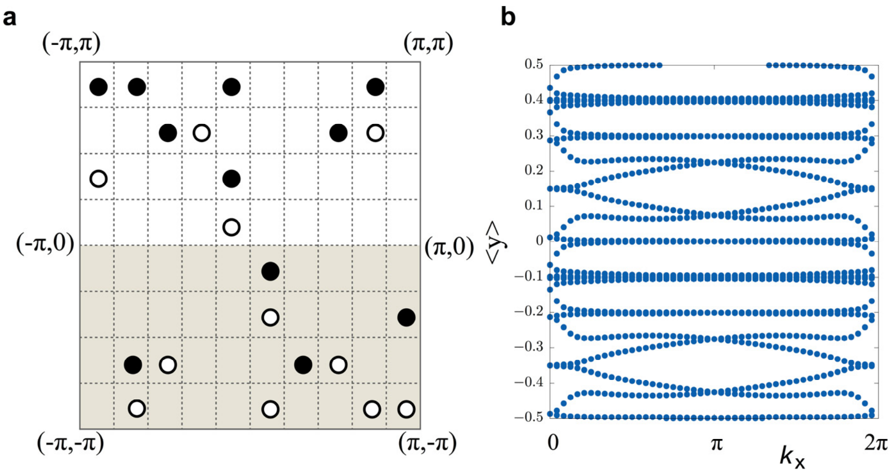

Supplementary Figure 4: Topological characterization of monolayer 1*T*'-WTe$_2$. a, Z_2 topological invariant as an obstruction of smoothly defining the phase of wave function in half of the BZ. **b**, The evolution of the hybrid Wannier charge center <y> as a function of k_x.

The clear separation of the conduction and valence bands everywhere in the Brillouin zone (BZ) (Supplementary Figure 1c) allows us to define the Z_2 topological invariant as usual for this time-reversal symmetric system. Here we apply two different numerical algorithms to characterize the topology of this system, *i.e.* topological obstruction of smoothly defining the phase of the wave function and the hybrid Wannier charge center (Wilson loop).

The band topology of a nontrivial electronic system is defined by the berry curvature of the occupied bands that are separated by an energy gap from the high-energy sector. For topological systems without time-reversal symmetry, the Chern invariant can be viewed as an obstruction to smoothly defining the wave function throughout the entire BZ, which is a torus in 2D. With the time-reversal symmetry present, the time-reversal constraint confines the evaluation of the topological invariant to only half of the BZ, *e.g.* the grey area of the Supplementary Figure 4a. It was shown that the topological invariant is given as

$$D = \frac{1}{2\pi}\left[\oint_{\partial\tau} d\ell\ \mathrm{A}\,(k) - \int_{\tau} d\tau\ \mathcal{F}(k)\right] mod\ 2, \qquad (1)$$

where $A(k)$ and $\mathcal{F}(k)$ are the Berry connection and Berry curvature, respectively.

$$A(k) = i\sum_n < u_{k,n}|\nabla_k|u_{k,n} > \text{ and } \mathcal{F}(k) = \nabla_k \times A(k). \qquad (2)$$

$\partial\tau$ and τ are the corresponding boundary and the area of the grey-color shaded region in Supplementary Figure 4a. The above formula can be efficiently evaluated on a discrete lattice consisting of small plaquettes, see Supplementary Figure 4a. These plaquettes are obtained by discretizing the first BZ with $k_i = b_i/N_i * i$ for $i = 0, \ldots, N_{i-1}$, where b_i is the

reciprocal vector and N_i is the number of slices along b_i. As b_1 and b_2 are not necessarily orthogonal, the resulting plaquette does not have to be a square. Along the closed edges of each plaquette, we calculated the phase change of the wave functions. The total phase change in half of the BZ yields the Z_2 topological invariant. If the total phase change is odd times of π, the system is topologically nontrivial. Otherwise, it is topologically trivial. In each plaquette, Equation (1) takes strictly integer value which is called *n*-filed value, they can be zero (empty), 1 (solid circle) or -1 (empty circle). The Z_2 topological invariant is given by (# of solid circle + # of empty circle) modulo 2.

An equivalent way of charactering a Z_2 topological insulator is to calculate the hybrid Wannier charge center, as shown in Supplementary Figure 4b. The nontrivial connectivity of the Wannier center (symbols), *i.e.* the *n*th curve connects upwards with the *(n+1)*th curve and downwards with the *(n-1)*th curve, characterizes the topology of the system. While the trivial band insulator is qualitatively different from the above picture, where every curve is separated from the others.

Supplementary Note 5. Topological edge states of a ribbon along *b*-edge and along *a*-edge with different edge termination

The detailed picture of the ES largely depends on the edge geometry in monolayer 1*T*'-WTe_2, which has a strong implication to the experimental detection of the topological density of states. The topological character, determined by the bulk band structure, is unaffected by the different choices of edge geometry. However, the different edge terminations may shift ES in energy, as shown in Supplementary Figure 5 (along the *b*-edge

of the unit cell shown in Supplementary Figure 1a) and Supplementary Figure 6a (along the top and bottom *a*-edge of the unit cell shown in Supplementary Figure 6b). The actual edge termination can be much more complicated than the cases studied in Fig. 1 and Supplementary Figure 6b. As a result, the topological ES measured at different spots of the step edge can appear at different binding energies.

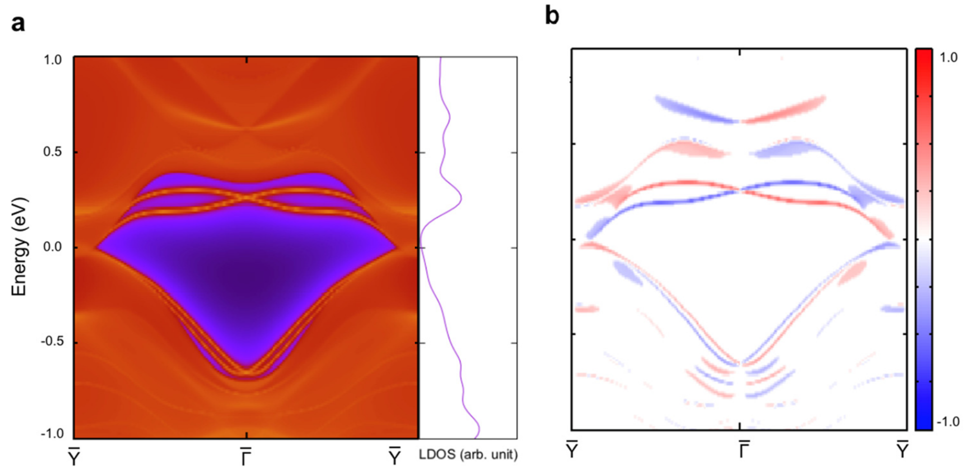

Supplementary Figure 5: Topological edge state along *b* direction. The topological ES spectra, the LDOS obtained by its momentum integration (**a**) and their corresponding spin z-components (**b**) of the monolayer 1*T*'-WTe_2 along the *b*-edge of the unit cell shown in Supplementary Figure 1a.

To compare with the experimentally measured ES, we integrate the calculated ES spectra over momentum, which give rise to the LDOS containing the ES and partial bulk

states (Supplementary Figure 5a, Supplementary Figure 6a and Supplementary Figure 7). Indeed, different edge configuration gives different LDOS of ES. It is seen from Supplementary Figure 7 that the two peaks at around 0 meV and 80 meV qualitatively resemble those of the measured ES of Point 1 and 2 in Supplementary Figure 2c.

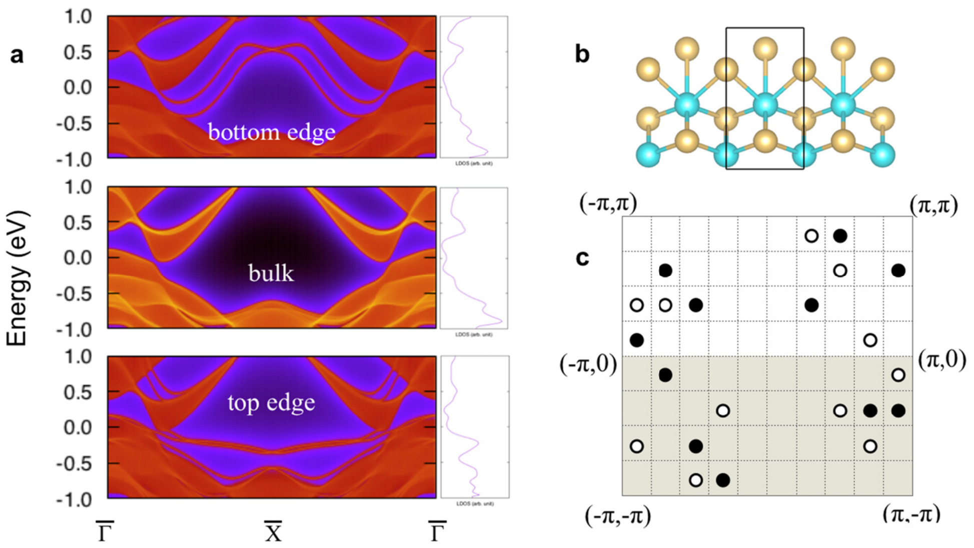

Supplementary Figure 6: Topological edge state along a direction with different terminations. The ES spectra, the corresponding LDOS (**a**) and the topological character (**c**) of monolayer 1*T*'-WTe_2 with different edge terminations shown in **b**. In **b** the top and bottom edges are of different geometries yielding the different binding-energy locations of the ES. Both ES in **a** are topologically nontrivial, which are proven by the *n*-field configuration in **c** as a topological obstruction to smoothly defining the wave function within half of the 2D BZ (*e.g.* the grey area).

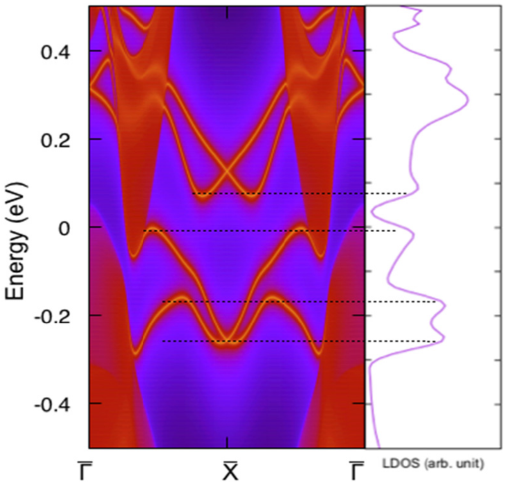

Supplementary Figure 7: LDOS of topological edge state. The topological ES spectra and the corresponding LDOS of monolayer 1*T*'-WTe_2 along the edge shown in Fig. 4a inset of main text. The dashed lines mark the correspondence of the extrema in ES and the peaks in LDOS.

Supplementary Note 6. Effect of external pressure on the QSH phase

1*T*'-WTe_2 becomes superconducting upon applying external pressure. Lattice constants ***a*** and ***b***, thus the in-plane geometry, are less changed by pressure, however ***c*** is largely reduced. The emergence of superconductivity demands a significant change of the states around the Fermi level, thus the electronic structure at low energy sector where the nontrivial band topology resides is expected to be modified by pressure. The question as to whether the topology of the monolayer 1*T*'-WTe_2 survives in this case naturally arises.

To construct the monolayer 1*T*'-WTe_2 under pressure, we extracted the lattice constants of the bulk 1*T*'-WTe_2 at 5.36 GPa and 10.934 GPa from Ref. 30 and fully relaxed

the internal coordinates of every atom. From the converged structure, we extracted one layer of WTe_2 as the monolayer and added sufficient vacuum in the ***c***-direction. We kept the relative position of each atom to be the fully relaxed position of the bulk. Repeating the electronic structure calculation and the topological analysis explained before, we found that, at the two pressures studied, the topological character of the monolayer 1*T*'-WTe_2 is unchanged, *i.e.* the systems remain as QSH semimetals. This conclusion is kept unchanged even after we further relaxed the internal coordinates of each atom in the monolayer. This is expected, as the interlayer coupling of WTe_2 is weak and the change of ***a*** and ***b*** is negligibly small. In Supplementary Table 1 and Supplementary Table 2, we give the coordinates of each atom in bulk and in monolayer geometry used in our calculations.

**bulk**	**Monolayer from bulk**	**Monolayer fully relaxed**
WTe_2 5.36 GPa bulk<br>1.00<br>6.157000 0.0000 0.0000<br>0.000000 3.4060 0.0000<br>0.000000 0.0000 13.2479<br>W Te<br>4 8<br>Direct<br>0.89891 0.5000 0.00068<br>0.10109 0.0000 0.50068<br>0.54122 0.0000 0.98457<br>0.45878 0.5000 0.48457<br>0.29240 0.5000 0.10273<br>0.70761 0.0000 0.60273<br>0.79987 0.0000 0.15205<br>0.20014 0.5000 0.65205<br>0.35473 0.0000 0.33315<br>0.64527 0.5000 0.83315<br>0.85268 0.5000 0.38267<br>0.14732 0.0000 0.88267	WTe_2 5.36 GPa mon. from bulk<br>1.0<br>6.1570 0.0000 0.0000<br>0.0000 3.4060 0.0000<br>0.0000 0.0000 52.992<br>W Te<br>2 4<br>Direct<br>0.10109 0.0000 0.12517<br>0.45878 0.5000 0.12114<br>0.70761 0.0000 0.15068<br>0.20013 0.5000 0.16301<br>0.35473 0.0000 0.08329<br>0.85268 0.5000 0.09567	WTe_2 5.36 GPa mon. relaxed<br>1.0000<br>6.15700 0.00000 0.00000<br>0.00000 3.40600 0.00000<br>0.00000 0.00000 52.9920<br>W Te<br>2 4<br>Direct<br>0.10011 0.00000 0.12512<br>0.45816 0.50000 0.12121<br>0.70842 0.00000 0.15191<br>0.20137 0.50000 0.16339<br>0.35699 0.00000 0.08292<br>0.84997 0.50000 0.09441

Supplementary Table 1: Crystal structure of bulk and monolayer 1*T*'-WTe_2 at 5.36 GPa.

bulk	Monolayer from bulk	Monolayer fully relaxed
WTe_2 10.934 GPa bulk<br>1.00000<br>6.12200 0.00000 0.00000<br>0.00000 3.33500 0.00000<br>0.00000 0.00000 12.78999<br>W Te<br>4 8<br>Direct<br>0.88343 0.50000 0.00067<br>0.11657 0.00000 0.50067<br>0.52217 0.00000 0.98459<br>0.47783 0.50000 0.48459<br>0.27288 0.50000 0.10609<br>0.72712 0.00000 0.60609<br>0.78244 0.00000 0.15708<br>0.21756 0.50000 0.65708<br>0.36706 0.00000 0.32819<br>0.63294 0.50000 0.82819<br>0.86865 0.50000 0.37923<br>0.13135 0.00000 0.87923	WTe_2 10.934 GPa mon. from bulk<br>1.0<br>6.12200 0.00000 0.00000<br>0.00000 3.33500 0.00000<br>0.00000 0.00000 51.15998<br>W Te<br>2 4<br>Direct<br>0.11657 0.00000 0.12517<br>0.47783 0.50000 0.12115<br>0.72711 0.00000 0.15152<br>0.21756 0.50000 0.16427<br>0.36706 0.00000 0.08205<br>0.86865 0.50000 0.09481	WTe_2 10.934 GPa mon. relaxed<br>1.0<br>6.12200 0.00000 0.00000<br>0.00000 3.33500 0.00000<br>0.00000 0.00000 51.15998<br>W Te<br>2 4<br>Direct<br>0.11571 0.00000 0.12507<br>0.47568 0.50000 0.12124<br>0.72704 0.00000 0.15358<br>0.21947 0.50000 0.16515<br>0.37245 0.00000 0.08117<br>0.86444 0.50000 0.09275

Supplementary Table 2: Crystal structure of bulk and monolayer 1*T*'-WTe_2 at 10.934 GPa.
